# Para-Markov chains and related non-local equations

**DOI:** 10.1007/s13540-025-00390-9

**Published:** 2025-04-02

**Authors:** Lorenzo Facciaroni, Costantino Ricciuti, Enrico Scalas, Bruno Toaldo

**Affiliations:** 1 Dipartimento di Scienze Statistiche, Sapienza Università di Roma, Rome, Italy; 2 Dipartimento di Matematica “Giuseppe Peano”, Università degli studi di Torino, Turin, Italy

**Keywords:** Fractional calculus operators, Non-Markovian dynamics, Semi-Markov chains, Schur lifetimes, Function of matrices, 26A33, 60G55, 60G09, 60G99

## Abstract

There is a well-established theory that links semi-Markov chains having Mittag-Leffler waiting times to time-fractional equations. We here go beyond the semi-Markov setting, by defining some non-Markovian chains whose waiting times, although marginally Mittag-Leffler, are assumed to be stochastically dependent. This creates a long memory tail in the evolution, unlike what happens for semi-Markov processes. As a special case of these chains, we study a particular counting process which extends the well-known fractional Poisson process, the last one having independent, Mittag-Leffler waiting times.

## Introduction

Continuous-time semi-Markov chains are obtained from Markov chains by relaxing the assumption of exponential waiting times. Several papers were devoted to chains with Mittag-Leffler distributed waiting times, among which the well-known fractional Poisson process is included (the reader can consult e.g. [2, 3, 5, 11, 16, 23, 25, 28–31, 34, 39]. A Mittag-Leffler distribution is characterized by a parameter $\nu \in (0,1]$, such that if $\nu =1$ one re-obtains the exponential distribution and a Markov chain, while, for $\nu \in (0,1)$, the distribution has a density $\psi$ with asymptotic power-law decay, $\psi (t)\sim t^{-\nu -1}$. In the latter case, the waiting times have infinite mean and variance, which is useful in models of anomalous diffusion (see e.g. [32]) as well as in financial applications (see e.g. [40]).

In the Markovian case, the transition matrix *P*(*t*) solves the Kolmogorov equation

$$\begin{aligned} \frac{d}{dt}P(t) = G P(t) \end{aligned}$$

where *G* is the infinitesimal generator of the chain. For semi-Markov chains with Mittag-Leffler waiting times, this equation is replaced by

$$\begin{aligned} \frac{d^\nu }{dt^\nu }P(t) = G P(t) \end{aligned}$$

where $d^\nu /dt^\nu$ denotes the Caputo fractional derivative, see also [14, 22, 36, 38] for the case where $\nu$ depends on the current state of the process, which is applied to models of anomalous diffusion in heterogenerous media.

Note that in the Markovian case, corresponding to $\nu =1$, one has a “local” equation: the time derivative - a local operator - is consistent with the lack-of-memory property typical of Markov chains. Indeed, the connection between Markov processes and first order differential equation is well-established.

On the contrary, if $\nu \in (0,1)$ one has a “non-local” equation: the integral operator of Volterra type $d^\nu /dt^\nu$ contains a memory kernel.

A limitation of these models is as follows. Semi-Markov processes lose memory of the past at renewal times only, i.e. when the chain jumps. Therefore, the future evolution of a semi-Markov process is only influenced by the recent past, and not by the whole past history. In this sense, the time-fractional derivative only contains information on the recent past through the age (i.e. the time elapsed from the previous renewal event).

In general, the study of non-Markovian processes is difficult. Therefore, any progress in this direction is of potential interest to a wide community of mathematicians and applied scientists. One goal is then to use the apparatus of fractional operators and time-fractional equations to treat the long-memory tail of some non-Markovian processes (which are not semi-Markov).

Our contribution to this goal is to define a class of processes that we call *(fractional) para-Markov chains*. These processes have a property in common with semi-Markov ones: the marginal distribution of each waiting time is Mittag-Leffler. However, all the waiting times are stochastically dependent, hence the process keeps memory of the whole past history. We then extend the mathematical techniques typically used for semi-Markov chains, including the use of fractional operators, to such a class of non-Markovian processes. Eventually, we obtain a governing equation of the form

$$\begin{aligned} \frac{d^\nu }{dt^\nu }P(t) = -(-G)^\nu P(t). \end{aligned}$$

What makes these processes analytically tractable is that, in distribution, they are proved to be a suitable mixture of Markov processes, hence the choice of the name *para-Markov*.

Our most general results concern the case of finite state space. However, we also deal with an important case with infinite countable state space, namely an extension of the fractional Poisson process. We refer to it as the *exchangeble fractional Poisson process*, for reasons that will become clear later. This is a counting process with unit jumps whose waiting times, although marginally having a Mittag-Leffler distribution, present an appropriate stochastic dependence. The latter is given by a particular Schur-type distribution (in the sense of [4, 7, 8]). Therefore, unlike the fractional Poisson process, our counting process is not a renewal process.

The structure of the paper is the following: Section 2 is devoted to some preliminaries on Markov and semi-Markov chains; Section 3 deals with the exchangeable fractional Poisson process; in Section 4 we introduce the general theory of para-Markov chains and study the finite-state case.

## Preliminaries

Let us consider a sequence of non-negative random variables $\theta = \{\theta _n\}_{n=1}^{\infty }$, which we interpret as the sequence of waiting times, and the stochastic process $T := \{T_n,\ n\in \mathbb {N}\}$ such that

$$\begin{aligned} T_n := \sum _{k = 1}^n \theta _k, \end{aligned}$$

with the convention $T_0 := 0$. Let ${\mathcal {S}}$ be a countable state space and let $Y = \{Y_n,\ n\in \mathbb {N}\}$ be a discrete-time stochastic process which takes value in ${\mathcal {S}}$. We say that the process $X = \{ X_t,\ t\ge 0 \}$, defined by

$$\begin{aligned} X_t = Y_n \qquad \qquad t\in [T_n, T_{n+1}),\ n\in \mathbb {N}, \end{aligned}$$

is a continuous-time chain.

We will consider three types of continuous-time chains, say Markov, semi-Markov, and para-Markov, the last one being introduced in this paper. In all three cases, the embedded chain *Y* is a discrete-time Markov chain, and thus what distinguishes one from the others is the joint distribution of the waiting times. Let $H = [h_{ik}]$ be the transition matrix of *Y*, such that

$$\begin{aligned} h_{ik} := \mathbb {P}[ Y_{n+1}=k | Y_n=i ] \qquad i,k \in {\mathcal {S}} \end{aligned}$$

under the convention $h_{ii}=0$.

Consider $\lambda : {\mathcal {S}} \rightarrow (0,\infty )$. The process *X* is a *continuous-time Markov chain* if (consult [33], page 94) the waiting times are such that

2.1 $$\begin{aligned} \mathbb {P}[\theta _1> t_1, \ldots , \theta _n > t_n | Y_0 = y_0,\ldots , Y_{n-1} = y_{n-1}]&= e^{-\lambda (y_0)t_1}\cdots e^{-\lambda (y_{n-1}) t_{n}}, \end{aligned}$$

i.e. the $\theta _i$s are conditionally independent, each of them having exponential distribution

2.2 $$\begin{aligned} \mathbb {P}[\theta _i > t | Y_{i-1} = x] = e^{-\lambda (x) t}. \end{aligned}$$

According to the above definition, the Markov chain is homogeneous in time.

A key object of continuous-time Markov chains is the generator of the process, denoted as $G = [g_{ij}],\ i,j\in S$. If $i\ne j$, then $g_{ij}$ represents the instantaneous rate at which the process moves from state *i* to state *j*, i.e. $g_{ij}=\lambda (i) h_{ij}$. Moreover, from  (2.2), the time spent in state *i* before transitioning to another state, is exponentially distributed with rate $-g_{ii} =\lambda (i)$. In compact form, we can write the generator as

2.3 $$\begin{aligned} g_{ij}= \lambda (i) (h_{ij}-\delta _{ij}), \end{aligned}$$

where $\delta _{ij}$ is the Kronecker delta.

The probability that the process is in state *j* at time *t*, given that it started in *i* at time 0, is denoted by $P_{ij}(t)$. We shall use the matrix form with $P(t) = [P_{ij}(t)]$. The family of operators $\{P(t),\ t\ge 0\}$ satisfies the semi-group property $P(t+s) = P(t)P(s)$ and it is the solution of the system of Kolmogorov backward (*a*) and forward (*b*) equation

2.4 $$\begin{aligned} (a){\left\{ \begin{array}{ll} d P(t)/dt = G P(t) \\ P(0) = I \end{array}\right. } \qquad \qquad (b){\left\{ \begin{array}{ll} d P(t)/ dt = P(t) G \\ P(0) = I \end{array}\right. }. \end{aligned}$$

*Semi-Markov chains* are obtained from Markov ones by relaxing the assumption of exponential waiting times. Then Equation  (2.2) is replaced by

2.5 $$\begin{aligned} \mathbb {P}[\theta _n > t | Y_{n-1} = x] = S_x(t), \end{aligned}$$

where $S_x(\cdot )$ is a generic survival function; this implies that the lack-of-memory property is satisfied only at time instants when jumps occur.

Let the distribution  (2.5) be absolutely continuous with density $f_x(\cdot )$. Moreover, let $p_{ij}(t)$ be the probability that the process *X* moves from state *i* to state *j* in a time interval [0, *t*], under the condition that 0 is a renewal time, and let $P=[p_{ij}(t)]$ be the transition matrix. The family $\{P(t), t\ge 0\}$ cannot satisfy the semigroup property, i.e. $P(t+s)\ne P(t)P(s)$ unless in the Markovian case where $S_x$ is exponential. By standard conditioning arguments, one can see that

2.6 $$\begin{aligned} p_{ij}(t) = \sum _{k\in S} h_{ik} \int _0^t f_i(\tau )\, p_{kj}(t-\tau ) d\tau + S_i(t)\, \delta _{ij}. \end{aligned}$$

Equation  (2.6) is called the semi-Markov renewal equation.

We are interested in semi-Markov chains whose waiting times follow the Mittag-Leffler distribution, which is defined by a particular choice of the survival function (2.5).

### Definition 1

A non-negative random variable *J* is said to follow a Mittag-Leffler distribution with parameters $\nu \in (0,1]$ and $\lambda \in (0, \infty )$ if

$$\begin{aligned} \mathbb {P}(J>t)= {\mathcal {M}}_{\nu }(-\lambda t^{\nu }), \;\; t \ge 0 \end{aligned}$$

where ${\mathcal {M}}_{\nu } (\cdot )$ is the one-parameter Mittag-Leffler function, defined by

2.7 $$\begin{aligned} {\mathcal {M}}_\nu (z) = \sum _{k=0}^\infty \frac{z^k}{\varGamma (1+\nu k)} \qquad z\in \mathbb {C}. \end{aligned}$$

Definition 1 gives an absolutely continuous distribution. So, consider semi-Markov chains whose waiting times are such that

2.8 $$\begin{aligned} \mathbb {P}[\theta _k > t | Y_{k-1} = x] = {\mathcal {M}}_{\nu }(-\lambda (x)t^{\nu }), \qquad x\in {\mathcal {S}}, \end{aligned}$$

i.e., conditionally to $Y_{k-1}=x$, the variable $\theta _k$ has Mittag-Leffler distribution with parameters $\nu$ and $\lambda (x)$. For $\nu =1$,  (2.8) reduces to an exponential distribution and hence the process becomes a continuous-time Markov chain, with generator *G* defined by $g_{ij}= \lambda (i) (h_{ij}-\delta _{ij})$. For $\nu \in (0,1)$ the process *X* is semi-Markov.

Moreover, it is known that such a process is governed by the following backward (*a*) and forward (*b*) fractional equations (for a proof sketch, which is based on the renewal equation  (2.6), see Proposition 2.1 in [38] and references therein):

2.9 $$\begin{aligned} (a){\left\{ \begin{array}{ll} d^\nu P(t)/dt^\nu = G P(t) \\ P(0) = I \end{array}\right. } \qquad \qquad (b){\left\{ \begin{array}{ll} d^\nu P(t)/dt^\nu = P(t) G \\ P(0) = I \end{array}\right. }. \end{aligned}$$

Note that the state space is not required to be finite. As a particular case, the fractional Poisson process is obtained by setting $\lambda (x) =\lambda$ for any $x\in {\mathcal {S}}$ and $h_{i,j}=1$ if $j = i+1$ and $h_{ij} = 0$ otherwise, whence *G* is such that $g_{ii}= -\lambda$ and $g_{i,i+1}=\lambda$. Therefore, considering the forward system *(b)* and setting $p_{0j}(t):=p_j(t)$, we obtain the governing equation often reported in the literature:

2.10 $$\begin{aligned} \frac{d^\nu }{dt^\nu } p_j(t) = -\lambda p_j(t)+\lambda p_{j-1}(t) \qquad p_j(0)= \delta _{j0} , \end{aligned}$$

where we set $p_{-1}(t) = 0$ for all $t\ge 0$, since the process takes non-negative values. There are many extensions of the fractional Poisson process, such as the time inhomogeneous extensions defined in [6, 26], as well as the counting processes studied in [12, 18, 27].

## The Exchangeable fractional Poisson process

The well-known fractional Poisson process has independent, Mittag-Leffler waiting times between arrivals. The goal here is to build a counting process which, in analogy to the fractional Poisson process, increases by 1 unit when an event occurs and each waiting time has marginal Mittag-Leffler distribution. However, as shown in the following definition, we relax the hypothesis of independence between the waiting times. This is useful in non-Markovian models, where one has counting processes that are not of renewal type.

### Definition 2

Let $\{J_k\}_{k=1}^\infty$ be a sequence of non-negative dependent random variables, such that, for all $n\in \mathbb {N}\setminus \{0\}$ we have

3.1 $$\begin{aligned} \mathbb {P}[J_1> t_1,\ \ldots ,\ J_n > t_n] = {\mathcal {M}}_{\nu }\left( -\lambda ^{\nu } \left( \sum _{k=1}^n t_k\right) ^{\nu }\right) \qquad \nu \in (0,1],\ \ \lambda \in (0,\infty ), \end{aligned}$$

where $t_k\ge 0,\ k \in \{1,\ldots ,n\}$. Moreover let $T_n:= \sum _{k=1}^nJ_k$, with the convention $T_0 := 0$. Then the process $N = \{N_t,\ t\ge 0\}$ defined by

$$\begin{aligned} N_t = n \qquad \qquad t\in [T_n, T_{n+1}) \end{aligned}$$

is said to be *exchangeable fractional Poisson process* with parameters $\lambda$ and $\nu$.

We note that each $J_k$ follows a marginal Mittag-Leffler distribution with parameters $\lambda ^\nu$ and $\nu$, in the sense of Definition 1; this can be obtained from formula (3.1) with $t_j = 0$ for each $j\ne k$. Another important feature is that the above sequence of waiting times is an infinite *Schur-constant* sequence. We recall that a sequence $\{X_k\}_{k=1}^\infty$ of non-negative random variables is said to be an infinite *Schur-constant* sequence if, for any $n\in \mathbb {N}\setminus \{0\}$, we have $\mathbb {P}(X_1>t_1, X_2>t_2, \dots , X_n>t_n)= S(t_1+t_2+\dots + t_n)$, for a suitable function *S* which does not depend on *n*. This is a particular model of *exchangeable* waiting times, in the sense that *S* depends on the $t_k$ through their sum only, whence the name we have chosen for our counting process; this feature makes the process easily tractable from a statistical point of view, see [4, 7, 8], and has several applications, e.g. in finance and insurance ([9, 41]). We further observe that for $\nu = 1$ we have that ${\mathcal {M}}_{1}(x)= e^x$ and (3.1) has the form

3.2 $$\begin{aligned} \mathbb {P}[J_1> t_1,\ \ldots ,\ J_n > t_n] = e^{- \lambda \sum _{k=1}^n t_k}, \end{aligned}$$

namely the waiting times are i.i.d. exponential and $N_t$ is a Poisson process of parameter $\lambda$. For $\nu < 1$ we go beyond the exponential case.

### Remark 1

From the joint survival function (3.1) it is possible to obtain the joint distribution function. Indeed, by observing that

$$\begin{aligned} \left\{ J_1 \le t_1, \ldots , J_n \le t_n \right\} ^{c} = \left\{ \{J_1> t_1\}\cup \cdots \cup \{J_n > t_n\} \right\} , \end{aligned}$$

by Poincaré Theorem we have

$$\begin{aligned}  &   \mathbb {P}[J_1 \le t_1, \ldots , J_n \le t_n] \\  &   \quad = 1 - \sum _{i } \mathbb {P}[J_i> t_i] + \sum _{ i< j} \mathbb {P}[J_i> t_i, J_j> t_j] + \cdots + (-1)^n \mathbb {P}[J_{1}> t_1, \ldots , J_{n} > t_n] \\  &   \quad = 1 - \sum _{i } {\mathcal {M}}_{\nu } (-\lambda ^\nu t_i^\nu ) + \sum _{ i< j} {\mathcal {M}}_{\nu }\left( -\lambda ^{\nu } \left( t_i+t_j\right) ^{\nu }\right) + \dots \\    &   \qquad + (-1)^n {\mathcal {M}}_{\nu }\left( -\lambda ^{\nu } \left( \sum _{k=1}^n t_k\right) ^{\nu }\right) \end{aligned}$$

where we have applied (3.1) to each term of the summation. Moreover, we also get the joint density as

3.3 $$\begin{aligned} f(t_1\ldots , t_n) = (-1)^n \frac{\partial ^n}{\partial t_1 \cdots \partial t_n} {\mathcal {M}}_{\nu }\left( -\lambda ^{\nu } \left( \sum _{k=1}^n t_k\right) ^{\nu }\right) . \end{aligned}$$

### Remark 2

By using $f(t_1, \dots , t_n)$, we can obtain the density of $T_n$, the time of the *n*-th jump:

3.4 $$\begin{aligned} f_{T_n}(u)&= \frac{d}{du} \mathbb {P}[T_n \le u ] \nonumber \\&= \frac{d}{du} \mathbb {P}\left[ \sum _{k = 1}^n J_k \le u \right] \nonumber \\&= \frac{d}{du} \int _{t_1+t_2+ \dots t_n \le u} f(t_1, \dots , t_n) dt_1\dots dt_n \nonumber \\&= \frac{(-1)^n}{\varGamma (n)} u^{n-1} {\mathcal {M}}^{(n)}_{\nu } (-\lambda ^\nu u^\nu ) \qquad u>0, \end{aligned}$$

where we used Equation (3.3). This can be seen as a generalization of the Erlang distribution that is recovered for $\nu =1$.

Before discussing some properties of the exchangeable fractional Poisson process, we need to recall the following definition (see [21, 24, 35]).

### Definition 3

A non-negative random variable *L* follows a Lamperti distribution of parameter $\nu \in (0,1]$ if its Laplace transform is given by

3.5 $$\begin{aligned} \mathbb {E}\left[ e^{-\eta L}\right] = {\mathcal {M}}_{\nu }\left( -\eta ^{\nu }\right) , \qquad \eta \ge 0. \end{aligned}$$

### Remark 3

1. For $\nu = 1$ we get $\mathbb {E}\left[ e^{-\eta L}\right] = e^{-\eta }$ which implies $L = 1$ almost surely.
2. For $\nu \in (0,1)$ then *L* is absolutely continuous with density given by
  3.6 $$\begin{aligned} f(t) = \frac{\sin (\pi \nu )}{\pi }\frac{t^{\nu - 1}}{t^{2\nu } + 2t^{\nu } \cos (\pi \nu ) + 1} \ \ \ \ \ \ t > 0. \end{aligned}$$
  One can see Equations (1.1) and (3.3) in [21] with $\theta = 0$ and $\eta = z^{\frac{1}{\alpha }}$ for details.

The following theorem shows that the exchangeable fractional Poisson process is equal in distribution to a time-changed Poisson process. This time-change consists in a random scaling of time based on a Lamperti variable.

### Theorem 1

Let $Q = \{Q_t,\ t\ge 0\}$ be a Poisson process with intensity $\lambda$ and $N = \{N_t,t\ge 0\}$ be the exchangeable fractional Poisson process, with parameters $\lambda$ and $\nu$. Let *L* have Lamperti distribution with parameter $\nu$. Then we have

$$\begin{aligned} N_t \overset{d}{=}\ Q_{Lt},\quad \forall t \ge 0, \end{aligned}$$

where $\overset{d}{=}$ denotes equality of finite dimensional distributions.

In Section 4 below, we will provide the proof of Theorem 4, which includes Theorem 1 as a particular case. For this reason, here we omit the proof of Theorem 1.

Once again, we stress that for $\nu = 1$ we have $L = 1$ almost surely, that is the time parameter $Lt = t$ is no longer stochastic, obtaining the Poisson case as a special case of exchangeable fractional Poisson process.

The equivalence in distribution of Theorem 1 leads to the governing equation of the process. We shall use the notation $\mathbb {P}[N_t = k ] =: p_k(t)$.

### Theorem 2

Let *N* be the exchangeable fractional Poisson process as in Definition 2. Then

3.7 $$\begin{aligned} \frac{d^\nu }{dt^\nu }p_k(t) = -\lambda ^{\nu } (I-B)^{\nu } p_k(t) \qquad p_k(0)= \delta _{k0} \end{aligned}$$

where *B* is the shift operator such that $Bp_k(t)=: p_{k-1}(t)$ and

$$\begin{aligned} (I-B)^{\nu } p_k(t) = \sum _{j = 0}^{\infty } \left( \begin{matrix} \nu \\ j \end{matrix}\right) (-1)^j B^j p_k(t)= \sum _{j = 0}^{\infty } \left( \begin{matrix} \nu \\ j \end{matrix}\right) (-1)^j p_{k-j}(t). \end{aligned}$$

### Proof

Recalling that the Poisson process $Q_t$ is such that

$$\begin{aligned} \mathbb {E}\left[ e^{-\eta Q_t}\right] = e^{-\lambda t ( 1 - e^{-\eta })} \qquad \eta \ge 0, \end{aligned}$$

and using Theorem 1 we have that $Q_{Lt}$ has the following moment generating function:

3.8 $$\begin{aligned} A(\eta , t)&= \mathbb {E}\left[ e^{-\eta Q_{Lt}}\right] \nonumber \\&= \int _0^\infty e^{-\lambda t ( 1 - e^{-\eta })l} \mathbb {P}(L\in dl)\nonumber \\&= \mathbb {E}\left[ e^{-t\lambda (1-e^{-\eta }) L } \right] \nonumber \\&= {\mathcal {M}}_{\nu }(-\lambda ^{\nu }(1-e^{-\eta })^{\nu }t^{\nu }). \end{aligned}$$

Given that $t\mapsto {\mathcal {M}}_\nu (ct^{\nu })$ is an eigenfunction of the Caputo derivative $d^{\nu }/dt^{\nu }$ with eigenvalue *c*, we get

3.9 $$\begin{aligned} \frac{d^{\nu }}{dt^{\nu }} A(\eta ,t) = -\lambda ^{\nu }\left( 1-e^{-\eta }\right) ^{\nu } A(\eta , t) \qquad A(\eta , 0) = 1. \end{aligned}$$

Using the equality

$$\begin{aligned} (1 - e^{-\eta })^{\nu } = \sum _{j = 0}^{\infty } \left( \begin{matrix} \nu \\ j \end{matrix}\right) (-1)^j e^{-\eta j} \end{aligned}$$

and applying the inverse Laplace transform in $\eta$ on both sides of Equation (3.9) we get the thesis. Indeed, it is possible to prove that $d^\nu p_k(t)/dt^\nu$ is well posed, by using similar arguments as in point (3) of the proof of Theorem 5 : despite the state space is infinite, the right hand side of equation (3.7) actually has a finite number of addends as in Theorem 5, because

$$\begin{aligned} (I-B)^\nu p_k(t)=\sum _{j = 0}^{k} \left( \begin{matrix} \nu \\ j \end{matrix}\right) (-1)^j p_{k-j}(t). \end{aligned}$$

$$\square $$

Before giving a final result, we recall the formula by Faà di Bruno that generalizes the chain rule for the *n*-th derivative of a function composition, see [13]. For *f*, *u* satisfying appropriate regularity conditions, we have

3.10 $$\begin{aligned} \left( \frac{d}{dx}\right) ^{n} f(u(x)) = n! \sum _{k = 1}^n \frac{f^{(k)}(u(x))}{k!} \sum _{h_1+\cdots +h_k = n} \prod _{i = 1}^k\frac{u^{(h_i)}(x)}{h_i!} \end{aligned}$$

where the second sum is over all *k*-tuples of non-negative integers $(h_1,\ldots , h_k)$ satisfying the constraint $\sum _{i = 1}^kh_i = n.$

We also recall that the *n*-th derivative of the Mittag-Leffler function ${\mathcal {M}}_\nu (z)$ is given by [15, 37]

3.11 $$\begin{aligned} \left( \frac{d}{dz}\right) ^{n} {\mathcal {M}}_\nu (z) = n! {\mathcal {M}}^{n+1}_{\nu ,n \nu +1} (z), \end{aligned}$$

where ${\mathcal {M}}^\gamma _{\alpha ,\beta } (z)$ is the Prabhakar function or three-parameter Mittag-Leffler function defined as

3.12 $$\begin{aligned} {\mathcal {M}}^{\gamma }_{\alpha ,\beta } (z) = \frac{1}{\varGamma (\gamma )} \sum _{k=0}^\infty \frac{\varGamma (k+\gamma ) z^k}{k! \varGamma (\alpha k + \beta )}. \end{aligned}$$

In the following theorem we find the explicit expression of $p_n(t)$ that solves the governing equation (3.7).

### Theorem 3

Let us consider the exchangeable fractional Poisson process *N* as in Definition  2. Then the marginal distribution of $N_t$, $p_n(t) = \mathbb {P}(N_t =n)$, is given by

3.13 $$\begin{aligned} p_n(t) = {\left\{ \begin{array}{ll} {\mathcal {M}}_{\nu }(-\lambda ^{\nu }t^{\nu }) &  n= 0 \\ \sum _{k = 1}^n (-1)^{(n+k)} (\lambda t)^{k \nu } {\mathcal {M}}_{\nu , k\nu + 1}^{k+1}(-\lambda ^{\nu }t^{\nu }) c(k,n;\nu ) &  n\ge 1 \end{array}\right. } \end{aligned}$$

with

$$\begin{aligned} c(k,n;\nu ) = \sum _{h_1 + \cdots + h_k = n} \prod _{i = 1}^k\frac{(\nu )_{h_i}}{h_i!} \end{aligned}$$

where $(\nu )_h := \nu (\nu - 1)\cdots (\nu - h +1)$ and the sum is over all *k*-tuples of non-negative integers $(h_1,\ldots , h_k)$ satisfying the constraint $\sum _{i = 1}^kh_i = n.$

### Proof

Theorem 1 guarantees that *N* is equal in distribution to a Poisson process with stochastic time parameter *Lt*, where *L* follows the Lamperti distribution of parameter $\nu$. Then, by conditioning, we have

3.14 $$\begin{aligned} p_n(t)&= \int _0^{\infty } \frac{e^{-\lambda l t}}{n!}\left( \lambda l t\right) ^n \mathbb {P}(L\in dl) \nonumber \\&= \frac{t^n}{n!}(-1)^n\left( \frac{d}{dt}\right) ^n\int _0^{\infty }e^{-\lambda lt} \mathbb {P}(L\in dl) \nonumber \\&= \frac{t^n}{n!}(-1)^n\left( \frac{d}{dt}\right) ^n \mathbb {E}\left[ e^{-\lambda t L}\right] \nonumber \\&= \frac{t^n}{n!}(-1)^n\left( \frac{d}{dt}\right) ^n {\mathcal {M}}_{\nu }\left( -\lambda ^{\nu } t^{\nu }\right) \qquad \qquad n\in \mathbb {N},\ t\ge 0 \end{aligned}$$

where we used (3.5). For $n = 0$ we immediately get the thesis. For $n\ge 1$ we can now use formulae (3.10) and (3.11), to get

$$\begin{aligned} p_n(t)&= \frac{t^n}{n!}(-1)^n n! \sum _{k =1}^n {\mathcal {M}}_{\nu , k\nu + 1}^{k+1} (-\lambda ^{\nu }t^{\nu }) \sum _{h_1 +\cdots + h_k = n} (-1)^k \lambda ^{k\nu } \prod _{s = 1}^k\frac{(t^{\nu })^{(h_s)}}{h_s!}\\&= (-1)^n t^n \sum _{k =1}^n {\mathcal {M}}_{\nu , k\nu + 1}^{k+1} (-\lambda ^{\nu }t^{\nu }) t^{k\nu - n}\sum _{h_1 +\cdots + h_k = n} (-1)^k \lambda ^{k\nu } \prod _{s = 1}^k\frac{(\nu )_{h_s}}{h_s!}\\&= \sum _{k = 1}^n (-1)^{(n+k)} (\lambda t)^{k \nu } {\mathcal {M}}_{\nu , k\nu + 1}^{k+1}(-\lambda ^{\nu }t^{\nu }) c(k,n;\nu ). \end{aligned}$$

$$\square $$

### Remark 4

An alternative proof of the previous theorem, also based on formulae (3.10) and (3.11), is now proposed. Firstly, starting from Equation (3.8), we have that the probability generating function of $N_t$ is given by

3.15 $$\begin{aligned} \mathbb {E}\left[ u^{N_t}\right]&= {\mathcal {M}}_{\nu } \left( -\lambda ^{\nu }t^{\nu } (1-u)^{\nu } \right) , \qquad |u|\le 1. \end{aligned}$$

Hence

3.16 $$\begin{aligned} p_n(t) = \frac{1}{n!} \left( \frac{d}{dt} \right) ^n {\mathcal {M}}_{\nu }(-\lambda ^{\nu }t^{\nu } (1-u)^{\nu }) \bigg |_{u = 0} \end{aligned}$$

which, by applying (3.10) and (3.11), coincides with Equation (3.13).

### Simulations

**Fig. 1:**
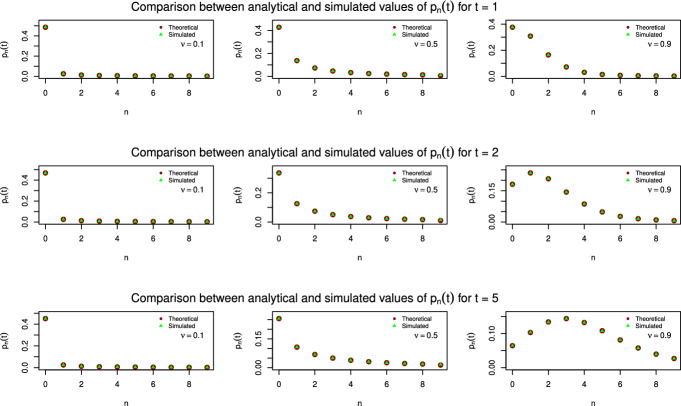
Comparison between analytical values of $p_n(t)$ obtained with formula (3.13) and corresponding simulated values, for $t = 1,2,5$ and $\nu = 0.1, 0.5, 0.9$

This paragraph presents some results concerning the numerical and Monte Carlo simulations of the analytical formulas derived in the previous Section. We have used the software R and the libraries MittagLeffleR, kStatistics and stabledist. Specifically, Figure 1 shows the values of $$ p_n(t) $$ for $$ n \in \{0, \ldots , 9\} $$ of the exchangeable fractional Poisson process, obtained by using the analytical formula (3.13) (red dots) and the corresponding simulated values (green triangles), in the case $$\lambda =1$$. Notably, these results are essentially coinciding. For the Monte Carlo simulation, we have used $$1,000,000 $$ independent random numbers following the Lamperti distribution; to this aim, we used that a Lamperti random variable of parameter $$\nu $$ is equal in law to the ratio of two independent, positive, $$\nu $$-stable random variables (see [21] for details). Then we have generated the vector $$\textrm{N}$$, of length 1, 000, 000, whose *j*-th component is a realization of a Poisson random variable of parameter $$ t \textrm{L} [j]$$, being $$\textrm{L}$$ the vector which contains the realizations the Lamperti variable. Finally we computed the relative frequency of the events that approximates $$ p_n(t) $$ for each $$ n $$. For the computation of the analytical values, we have used the expression of (3.10) in terms of exponential Bell polynomials (see [10] for details). Specifically, used the formula (3.14), firstly computing a function of *n* and *t* which gives the value of the coefficient and then multiplying it by the *n*-th time derivative of the Mittag-Leffler with parameter $$-\lambda ^{\nu }t^{\nu }$$. We repeated the simulation for $$t = 1,2,5$$ and $$\nu = 0.1, 0.5, 0.9$$.

**Fig. 2:**
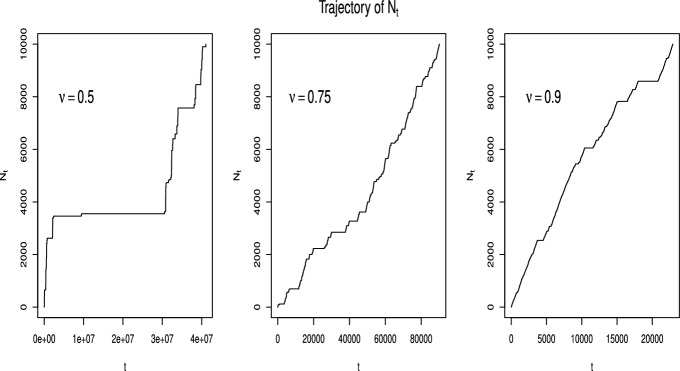
Trajectory of the exchangeable fractional Process $N_t$ for $\nu = 0.5, 0.75, 0.9$ and number of events $n\le 10000$

Figure 2 shows a trajectory of the exchangeable fractional Poisson process defined in Definition 2 for different values of the parameter $\nu$, up to $n = 10,000$ events. Here we have generated *n* values from a Lamperti distribution of parameter $\nu$, for $\nu = 0.5, 0.75, 0.9$, as explained before, and we have saved them in a vector L. Then we have generated the first *n* waiting times, each of them as a realization of an exponential distribution with parameter L[*j*], according to Theorem 1. Finally, the trajectory is obtained cumulating the waiting times. The waiting times become longer, as the value of $\nu$ decreases. Note that before performing both simulations, the seed was set to 1. The interested reader can find the code used to generate the figures at https://github.com/Lorenzo-Facciaroni/Exchangeable-fractional-Poisson

## Para-Markov chains in continuous-time

We here introduce para-Markov chains, which include the exchangeable fractional Poisson process as a notable case.

### Definition 4

Let $Y = \{Y_n,\ n\in \mathbb {N}\}$ be a discrete time Markov chain on a finite or countable state space ${\mathcal {S}}$. For $\nu \in (0,1]$ and $\lambda : {\mathcal {S}} \rightarrow (0,\infty )$, let $\{J_k\}_{k=1}^\infty$ be a sequence of non-negative random variables, such that, $\forall n\in \mathbb {N}\setminus \{0\}$,

4.1 $$\begin{aligned}&P(J_1>t_1, \dots , J_n>t_n |Y_0=y_0, \dots , Y_{n-1}=y_{n-1}) \nonumber \\&\quad = {\mathcal {M}}_\nu \left( -\left( \sum _{k=1}^{n} \lambda (y_{k-1})t_{k} \right) ^\nu \right) \end{aligned}$$

where $t_k\ge 0,\ k \in \{1,\ldots ,n\}$. A continuous-time chain $X = \{X_t,\ t\in [0, \infty )\}$ such that

$$\begin{aligned} X_t = Y_n \qquad \qquad t\in [T_n, T_{n+1}),\ n\in \mathbb {N} \end{aligned}$$

where $T_n:= \sum _{k=1}^nJ_k$ and $T_0 := 0$, is said to be a continuous-time para-Markov chain.

Note that if $\nu =1$ one re-obtains the joint survival function (2.1) and then the process is a continuous-time Markov chain. For $\nu \in (0,1)$ the above process is neither Markov nor semi-Markov, because of the dependence between waiting times $J_k$.

### Remark 5

We observe that Definition 4 completely defines the finite dimensional distributions of a para-Markov chain *X*. Indeed, denoting by $N_t$ the number of jumps up to time *t*, and letting $t_1<t_2<\dots <t_n$ we have

$$\begin{aligned}&\mathbb {P}\left[ \bigcap _{j=1}^n\{X_{t_j}=x_j\}\right] = \sum _{k_1 \le k_2\le \cdots \le k_n}^{\infty }\mathbb {P}\left[ \bigcap _{j=1}^n\{X_{t_j}=x_j\}, \, \bigcap _{j=1}^n\{N_{t_j}=k_j\}\right] \\&\quad = \sum _{k_1 \le k_2\le \cdots \le k_n}^{\infty }\mathbb {P}\left[ \bigcap _{j=1}^n\{Y_{N_{t_j}}=x_j\}, \, \bigcap _{j=1}^n\{N_{t_j}=k_j\}\right] \\&\quad = \sum _{k_1 \le k_2\le \cdots \le k_n}^{\infty }\mathbb {P}\left[ \bigcap _{j=1}^n\{Y_{k_j}=x_j\}, \, \bigcap _{j=1}^n\{T_{k_j} \le t_j< T_{k_j+1} \}\right] \\&\quad = \sum _{k_1 \le k_2\le \cdots \le k_n}^{\infty }\mathbb {P}\left[ \bigcap _{j=1}^n \left. \{T_{k_j} \le t_j < T_{k_j+1} \} \, \right| \, \bigcap _{j=1}^n\{Y_{k_j}=x_j\} \right] \mathbb {P}\left[ \bigcap _{j=1}^n\{Y_{k_j}=x_j\}\right] \end{aligned}$$

where, in principle, the last term can be computed by means of the matrix *H* of the embedded chain and the waiting time distribution given in Definition 4.

The reason for using the expression *para-Markov* is due to the following theorem. According to it, *X* is equal in distribution to a time-changed continuous-time Markov process. The time-change consists in rescaling the time *t* by a Lamperti random variable.

### Theorem 4

Let $M = \{M_t,\ t\in [0,\infty )\}$ be a continuous-time Markov chain defined by (2.1) and $X = \{X_t,\ t\in [0,\infty )\}$ be a para-Markov chain as in Definition 4. Let *L* be a Lamperti random variable, as in Definition  3. Then we have

$$\begin{aligned} X_t \overset{d}{=}\ M_{Lt} \qquad \qquad \forall t \ge 0, \end{aligned}$$

where $\overset{d}{=}$ denotes equality of finite dimensional distributions.

### Proof

Let $\{\theta _k\} _{k=1}^\infty$ be a sequence of exponential random variables as in (2.1). Then we have

$$\begin{aligned} M_t=Y_n, \qquad \tau _n \le t< \tau _{n+1}, \end{aligned}$$

where $\tau _n := \sum _{k=1}^n \theta _k$ and $\tau _0:=0$. By the random scaling of time $t \rightarrow Lt$ we have

$$\begin{aligned} M_{Lt}=Y_n, \qquad \frac{\tau _n}{L} \le t< \frac{\tau _{n+1}}{L}, \end{aligned}$$

which means that the *k*-th waiting time of $M_{Lt}$ is equal to

$$\begin{aligned} \frac{\theta _k}{L}=\frac{\tau _{k+1}-\tau _k}{L}. \end{aligned}$$

Thus, to prove that $M_{Lt}$ coincides with $X_t$ in the sense of finite-dimensional distributions, it is sufficient to show that the sequence of waiting times $\{\theta _k/L\} _{k=1}^\infty$ of $M_{Lt}$ has joint distribution given by (4.1) . This can be done using a conditioning argument, together with the above definition of Lamperti distribution:

$$\begin{aligned}  &   \mathbb {P}\biggl [\left. \frac{\theta _1}{L}> t_1,\ \ldots ,\ \frac{\theta _n}{L} > t_n \right| Y_0=y_0, \ldots Y_{n-1}=y_{n-1} \biggr ] \\  &   \quad =\int _0^{\infty }e^{-l \sum _{k=1}^n \lambda (y_{k-1})t_k}\mathbb {P} (L\in dl) \\  &   \quad = \mathbb {E}\left[ e^{- \left( \sum _{k=1}^n \lambda (y_{k-1}) t_k\right) L }\right] = {\mathcal {M}}_{\nu }\left( - \left( \sum _{k=1}^n \lambda (y_{k-1})t_k\right) ^{\nu }\right) . \end{aligned}$$

This completes the proof. $\square$

As explained before, Definition 4 of para-Markov process holds even with ${\mathcal {S}}$ a countable set. However, from now on, we shall consider the case of finite state space, say $|{\mathcal {S}}| = n \in \mathbb {N}$. Without loss of generality, we shall write ${\mathcal {S}} =\{ 1,\ldots , n \}$. In this scenario, the generator *G* of the Markov process *M* in Theorem 4 is an $n \times n$ matrix. Moreover, the systems of Kolmogorov backward and forward equations (2.4) have the following solution

$$\begin{aligned} P(t) = e^{Gt}. \end{aligned}$$

Furthermore, from Equation (2.3) the following decomposition holds in matrix from

$$\begin{aligned} G = \varLambda (H-I) \end{aligned}$$

being $\varLambda = \text {diag} \left( \lambda (1),\ldots , \lambda (n)\right)$ and *I* the identity matrix.

### Remark 6

The above considerations allow us to reinterpret Theorem 4 as follows. The transition matrix of the Markov chain *M* can be written as $P(t) = e^{\varLambda (H-I) t}$. Then changing time $t \rightarrow Lt$ is equivalent to replacing $\varLambda$ with $L \varLambda$, i.e. rescaling the time parameter is equivalent to rescaling the expectation of each waiting time.

The next theorem is the main result of the paper and gives us the governing equation of a para-Markov chain as well as its solution, written in matrix form.

For a matrix $A\in \mathbb {C}^{n\times n}$ we shall indicate with $\rho (A)$ the spectral radius of *A* and $\sigma (A)$ the spectrum. We use the natural norm $||\cdot ||: \mathbb {C}^{n\times n}\rightarrow \mathbb {R}^{+}$; this is a matrix norm induced by a vector norm, i.e.

$$\begin{aligned} ||A|| := \sup _{||x||_{v} = 1} ||Ax||_{v}, \end{aligned}$$

where $||\cdot ||_{v}:\mathbb {C}^n\rightarrow \mathbb {R}^{+}$ is a vector norm. Moreover, *A* is said to be convergent if there exists a natural norm such that

$$\begin{aligned} \lim _{k\rightarrow \infty } ||A^k|| = 0. \end{aligned}$$

We shall use the notation $\mathbb {C}^{-} := \{z\in \mathbb {C}\ s.t.\ \Re \{z\} < 0\}$.

For a scalar function $f:\mathbb {C}\rightarrow \mathbb {C}$, we refer to the meaning of *f*(*A*), being $A\in \mathbb {C}^{n\times n}$, as discussed in Chapter 1 of [19]. Specifically, let *A* have canonical Jordan decomposition $A = Z^{-1}JZ$, where *J* is the block diagonal matrix, while *Z* is the matrix whose columns contain the generalized eigenvectors. Hence $J = \textrm{diag}({J_{m_1}(\alpha _1), \ldots , J_{m_p}(\alpha _p}))$, where $J_{m_k}(\alpha _k)$ denotes a Jordan block with dimension $m_k$ corresponding to the eigenvalue $\alpha _k$, i.e. it has $\alpha _k$ on the diagonal and 1 above the diagonal; eigenvalues related to distinct blocks do not need to be distinct. For *f*(*A*) to be well defined, we need to require that $f(\cdot )$, as a scalar function, is defined on the spectrum of *A*, i.e. there must exist the derivatives

$$\begin{aligned} f^{(j)}(\alpha _k), \ \ \ j = 0,\ldots , n_k - 1,\ k = 1,\ldots , s \end{aligned}$$

with *s* the number of distinct eigenvalues of *A* and $n_k$ the order of the largest Jordan block where $\alpha _k$ appears. We say that $n_k$ is the *index* of $\alpha _k$. In this case we can use the Jordan canonical decomposition $A = Z^{-1}JZ$ to define

4.2 $$\begin{aligned} f(A) := Z^{-1} f(J) Z = Z^{-1} \text {diag}\left( f(J_{m_1}(\alpha _1)), \ldots , f(J_{m_p}(\alpha _p))\right) Z \end{aligned}$$

being *p* the number of Jordan blocks, i.e. the number of independent eigenvectors of *A*, and

$$\begin{aligned} f\left( J_{m_i} (\alpha _i)\right) := \left[ \begin{matrix} f(\alpha _i) &  f'(\alpha _i) &  \cdots &  \frac{f^{(m_i-1)}(\alpha _i)}{(m_i - 1)!} \\   &  f(\alpha _i) &  \ddots &  \vdots \\   &  &  \ddots &  f'(\alpha _i) \\   &  &  &  f(\alpha _i) \end{matrix}\right] . \end{aligned}$$

### Remark 7

Since the Mittag-Leffler function ${\mathcal {M}}_\nu$ defined in (2.7) is entire, then it is defined on the spectrum of any matrix $A \in \mathbb {C}^{n\times n}$. Let *A* have Jordan decomposition $A= Z^{-1}JZ$. Then, being $M_\nu$ defined by a power series, the matrix ${\mathcal {M}}_\nu (A)$ can be explicitly obtained as follows (see [15])

4.3 $$\begin{aligned} {\mathcal {M}}_\nu (A)&= \sum _{k=0}^\infty \frac{A^k}{\varGamma (1+\nu k)} = \sum _{k=0}^\infty \frac{\overbrace{Z^{-1}JZ\ Z^{-1}JZ \cdots \ Z^{-1}JZ}^{\text {k times}}}{\varGamma (1+\nu k)} \nonumber \\&= Z^{-1} \biggl ( \sum _{k=0}^\infty \frac{J^k}{\varGamma (1+\nu k)}\biggr ) Z = Z^{-1}{\mathcal {M}}_\nu (J)Z \end{aligned}$$

which coincides with expression given in (4.2). For $\nu = 1$, we have ${\mathcal {M}}_{\nu }(x)= e^x$ and thus we get the exponential of a matrix as

$$\begin{aligned} e^A = Z^{-1} e^J Z. \end{aligned}$$

In this case, to compute $e^J$ explicitly, we observe that each Jordan block can be decomposed as

$$\begin{aligned} J_{m_k} = \alpha _k I + N_k \end{aligned}$$

where *I* is the identity matrix and $N_k$ is nilpotent of order $m_k$. The matrices $\alpha _k I$ and $N_k$ commute and thus the *k*-th block is given by

$$\begin{aligned} e^{J_{m_k}} = e^{\alpha _k}e^{N_k} = e^{\alpha _k} \sum _{s=0}^{m_k-1} \frac{(N_k)^s}{s!}, \end{aligned}$$

i.e. it is sufficient to compute a finite sum.

Before stating the theorem, we recall that a matrix *A* is said to be *irreducible* if it is not similar via a permutation to a block upper triangular matrix, i.e. it does not have invariant subspaces. Indeed, if the generator of a continuous time Markov chain is irreducible, then there is a non-zero probability of transitioning from any state to any other state.

### Theorem 5

Let us consider a para-Markov chain *X* and the related Markov chain *M*, in the sense of Theorem 4, with generator *G*. Let $P(t)=[p_{ij}(t)]$ be the transition matrix of *X*, i.e. $p_{ij}(t)=\mathbb {P}(X_t=j|X_0=i),\ i,j\in {\mathcal {S}}$. If *G* is irreducible, then

1. The matrix $-(-G)^\nu$ exists for any $\nu \in (0,1]$,
2. The transition matrix has the form
  $$\begin{aligned} P(t) = {\mathcal {M}}_{\nu }(-(-G)^{\nu }t^{\nu }), \end{aligned}$$
3. *P*(*t*) is the solution of
  4.4 $$\begin{aligned} \frac{d^{\nu }}{dt^{\nu }} P(t) = -(-G)^{\nu } P(t) \end{aligned}$$
  with initial condition $P(0) = I$.

### Proof

Let us split the proof in three parts.

1. Since the function $f(x) = (-x)^{\nu },\ \nu \in (0,1]$, is not differentiable at $x=0$, then, according to (4.2), it is defined on the spectrum of *G* if either *G* does not have the eigenvalue 0 or *G* does have the eigenvalue 0 with index $n=1$. However, we shall see that *G* necessarily has the eigenvalue 0. Thus, we shall show that a sufficient condition for *G* to have the eigenvalue 0 with index 1 is its irreducibility; indeed, irreducibility of *G* implies that 0 is a simple eigenvalue, i.e. its algebraic multiplicity is 1.
  We indicate with ${\textbf{1}}$ the vector in $\mathbb {R}^{n}$ with all coordinates equal to 1. The row sums are 0 by (2.3), which gives
  $$\begin{aligned} G {\textbf{1}} = {\textbf{0}}. \end{aligned}$$
  It means that *G* has 0 as eigenvalue with correspondent eigenvector ${\textbf{1}}$.
  Moreover, we know that $g_{ij} \ge 0,\ i\ne j$ so, given the definition of the diagonal elements, we define
  $$\begin{aligned} R_{ii} := \sum _{j\ne i} |g_{ij}| = \sum _{j\ne i} g_{ij} = -g_{ii} \ \ \ i\in \{1,\ldots , n\} \end{aligned}$$
  which implies that the so-called Gershgorin discs $D(g_{ii}, R_{ii})= \{ z\in \mathbb {C}\ s.t. \ |z-g_{ii}|\le R_{ii} \}$ are subsets of $\mathbb {C}^{-} \cup \{0\}$. The Gershgorin theorem in [17] ensures that all the eigenvalues of *G* lie in the union of such discs, which, in our case, is contained in $\mathbb {C}^{-} \cup \{0\}$.
  Now, letting $\eta := \max \{g_{ij},\ i,j\in {\mathcal {S}}\}$ and considering that $\rho (G) > 0$, the matrix *T* defined by
  $$\begin{aligned} T := \frac{1}{\eta \rho (G)} G + I \end{aligned}$$
  is irreducible as well and it has non-negative entries. It follows by linearity of the eigenvalues that $\rho (T) = 1$ is an eigenvalue of *T*. Moreover, since the eigenvalues of *G* lie in $\mathbb {C}^{-} \cup \{0\}$, then all the eigenvalues of *T* lie in $D\left( \frac{1}{2}, \frac{1}{2} \right)$, which is the closed disc centered in $\frac{1}{2}+0i$ and radius $\frac{1}{2}$. The Perron-Frobenius theorem (see Paragraph 8.3 in [20]) guarantees that 1 is actually a simple eigenvalue and it is called the Perron-Frobenius eigenvalue. By applying the inverse formula $T\mapsto \eta \rho (G)(T-I)$, we get that $\alpha = 0$ is simple for *G*.
2. For a Lamperti random variable *L*, the function
  4.5 $$\begin{aligned} z \mapsto \int _{0}^\infty e^{ztl} \mathbb {P}(L\in dl) \end{aligned}$$
  is well defined for $\Re \{z\}\le 0$. Moreover (4.5) is analytic for $z\ne 0$, which is clear also by expressing it as
  4.6 $$\begin{aligned} z \mapsto \int _0^{\infty }e^{ztl}\mathbb {P}(L\in dl) = {\mathcal {M}}_\nu (-t^\nu (-z)^\nu ). \end{aligned}$$
  Such a function is well defined on the spectrum of *G*, since *G* has 0 as a simple eigenvalue by the irreducibility assumption, and furthermore all the other eigenvalues have negative real part. By virtue of this consideration, together with the time-change Theorem 4, *P*(*t*) takes the following matrix form in the sense of (4.2)
  $$\begin{aligned} P(t)&= \int _0^{\infty }e^{Gtl}\mathbb {P}(L\in dl)= {\mathcal {M}}_{\nu }(-(-G)^{\nu }t^{\nu }), \end{aligned}$$
  as desired.
3. Assume, for the moment, that $\frac{d^\nu }{dt^\nu }P(t)$ exists, it is continuous and Laplace transformable. To prove the statement, we preliminary recall that a square matrix *B* is convergent iff $\rho (B) < 1$; in this case $I-B$ is non-singular, such that
  4.7 $$\begin{aligned} (I - B)^{-1} = \sum _{k = 0}^{\infty } B^k. \end{aligned}$$
  Now, let us consider $g(t) = {\mathcal {M}}_{\nu }\left( -A t^{\nu }\right)$, being $A\in \mathbb {C}^{n\times n}$, and compute the Laplace transform
  4.8 $$\begin{aligned} \tilde{g}\left( s\right)&= \int _0^{\infty }e^{-st} {\mathcal {M}}_{\nu }\left( -A t^{\nu }\right) dt, \qquad s\in \mathbb {C}, \end{aligned}$$
  where the integral is meant component-wise. Being ${\mathcal {M}}_\nu$ entire, we have
  $$\begin{aligned} \tilde{g}\left( s\right)&= \int _0^{\infty }e^{-st}\sum _{k = 0}^{\infty } \frac{(-1)^kA^k t^{k\nu }}{\varGamma (1 + \nu k)}dt \\&= \frac{1}{s}\sum _{k = 0}^{\infty }\frac{(-1)^kA^k }{s^{\nu k}}\\&= \frac{1}{s}\sum _{k = 0}^{\infty }\left( \frac{-A }{s^{\nu }}\right) ^k \end{aligned}$$
  and then ${\tilde{g}}(s)$ converges for all *s* such that the spectral radius of $-A/s^\nu$ is less than 1, namely the Laplace transform is certainly defined for $\Re \{s\}>(\rho (A)) ^{1/\nu }$. Moreover, the Laplace inversion Theorem ensures that ${\tilde{g}}(s)$ is analytic in the same region $\Re \{s\}>(\rho (A)) ^{1/\nu }$ and thus uniquely identifies *g*(*t*). Using (4.7) we obtain
  4.9 $$\begin{aligned} \tilde{g}\left( s\right)&= \frac{1}{s}\left( I + \frac{A}{s^{\nu }}\right) ^{-1} \nonumber \\&= s^{\nu -1}\left( s^{\nu } I + A\right) ^{-1} \ \ \ \ \ \ \ \Re \{s\} > \left( \rho (A)\right) ^{\frac{1}{\nu }}. \end{aligned}$$
  We now look at the solution of the following problem
  4.10 $$\begin{aligned} \frac{d^{\nu }}{dt^{\nu }} h(t) = - A h(t) \qquad h(0) = I, \end{aligned}$$
  being $A\in \mathbb {C}^{n\times n}$. By applying the Laplace transform component-wise on both sides we get
  $$\begin{aligned} s^{\nu } \tilde{h}\left( s\right) - s^{\nu - 1} h(0)&= - A \tilde{h}\left( s\right) \qquad s\in \mathbb {C} \end{aligned}$$
  namely
  $$\begin{aligned} \left( I + \frac{A}{s^{\nu }}\right) \tilde{h}\left( s\right)&= s^{-1} I \qquad s\in \mathbb {C} \end{aligned}$$
  For $\Re \{s\} > \left( \rho (A)\right) ^{\frac{1}{\nu }}$, we have that $\left( I + \frac{A}{s^{\nu }}\right)$ is non-singular and then
  $$\begin{aligned} \tilde{h}\left( s\right) = s^{\nu -1}\left( s^{\nu } I + A\right) ^{-1} \ \ \ \ \ \ \ \Re \{s\} > \left( \rho (A)\right) ^{\frac{1}{\nu }} \end{aligned}$$
  which coincides with the Laplace transform (4.9). The inverse Laplace transform ensures equality for almost all $t>0$; moreover, continuity of $t\rightarrow P(t)$, which stems from the expression in point (2) of the present Theorem, ensures equality for all $t>0$. Hence $h(t) = {\mathcal {M}}_{\nu }\left( -A t^{\nu }\right)$ solves the problem (4.10). To conclude, we finally set $A = (-G)^{\nu }$.
  It remains to prove that $\frac{d^\nu }{dt^\nu }P(t)$ exists and it is continuous. The convolution $t\rightarrow \int _0 ^t \bigl (P(\tau )-P(0) \bigr ) \frac{(t-\tau ) ^{-\nu }}{\varGamma (1-\nu )} d\tau$ is well defined (see Prop. 1.6.4 in [1]). By using similar calculations as above (of which we omit the details), it is easy to prove that the two functions $t\rightarrow \int _0 ^t \bigl (P(\tau )-P(0) \bigr ) \frac{(t-\tau ) ^{-\nu }}{\varGamma (1-\nu )} d\tau$ and $t\rightarrow \int _0 ^t-(-G)^\nu P(\tau )d\tau$ have the same Laplace transform. Hence they coincide for almost all $t>0$. Moreover, both functions are continuous since *P*(*t*) is continuous by the expression given in point (2) of the Theorem. Hence the two functions coincide for any $t>0$:
  4.11 $$\begin{aligned} \int _0 ^t \bigl (P(\tau )-P(0) \bigr ) \frac{(t-\tau ) ^{-\nu }}{\varGamma (1-\nu )} d\tau = \int _0^t -(-G)^\nu P(\tau ) d \tau . \end{aligned}$$
  The right side of (4.11) is differentiable for $t>0$ because $-(-G)^{\nu }P(\tau )$ is component-wise continuous as it is a linear combination of continuous functions, and this is true also for the left side because the equality holds pointwise. Hence the Caputo derivative exists and is continuous. Now if we apply the time derivative to both sides, we obtain the desired equation.

$$\square $$

### Remark 8

By the above considerations, the matrix $(-G)^\nu$ is given by

$$\begin{aligned} (-G)^{\nu } = Z^{-1} \text {diag}\left( (J_{m_1}(\alpha _1))^{\nu }, \ldots , (J_{m_p}(\alpha _p))^{\nu }\right) Z \end{aligned}$$

where

$$\begin{aligned} \left( J_{m_i}(\alpha _i)\right) ^{\nu } = \left[ \begin{matrix} \alpha _i^{\nu } &  \nu \alpha _i^{\nu -1} &  \cdots &  \frac{(\nu )_{m_i-1}\alpha _i ^{\nu -m_i+1}}{(m_i-1)!} \\   &  \alpha _i^{\nu } &  \ddots &  \vdots \\   &  &  \ddots &  \nu \alpha _i^{\nu -1} \\   &  &  &  \alpha _i^{\nu } \end{matrix}\right] \end{aligned}$$

and one can see that the eigenvalue 0 must have index 1, being $z\mapsto (-z)^\nu$ not differentiable at 0 for $\nu \in (0,1)$.

### Remark 9

Equation (4.4) does not uniquely identify our para-Markov chain. For example, consider the process *M*(*H*(*L*(*t*))) where *M* is a Markov chain with generator *G*, *H* is a stable subordinator with index $\nu$ and *L* is an inverse stable subordinator with index $\nu$, under the assumption that *M*, *H* and *L* are independent. This process is governed by the same equation (4.4), even if it is not a para-Markov chain but a semi-Markov one.

## Final remarks

By using the same techniques as in the previous section, we find an interesting result on semi-Markov chains. Consider, indeed, semi-Markov chains with Mittag-Leffler waiting times recalled in Section 2, i.e. those governed by the equations (2.9).

### Proposition 1

If the state space ${\mathcal {S}}$ is finite, then the solution of (2.9) has the following matrix form

$$\begin{aligned} P(t) = {\mathcal {M}}_{\nu }(G t^{\nu }) . \end{aligned}$$

### Proof

It is sufficient to adapt the arguments used in the proof of point 3) of Theorem 5, setting $A = -G$. $\square$

To the best of our knowledge, the result in Proposition 1 is new. Indeed, in the literature, *P*(*t*) has been written by using the composition of the corresponding Markov process with an inverse stable subordinator (see e.g. [30]), but the explicit solution in matrix form has never been written.

**Table 1:** Comparison between continuous-time Markov, semi-Markov and para-Markov processes

	Governing Equation	Solution
Markov	$d P(t)/dt = G P(t)$	$P(t) = e^{Gt}$
semi-Markov	$d^{\nu } P(t)/dt^{\nu } = GP(t)$	$P(t) = {\mathcal {M}}_{\nu }\left( Gt^{\nu }\right)$
para-Markov	$d^{\nu } P(t) / dt^{\nu } = -(-G)^{\nu } P(t)$	$P(t) = {\mathcal {M}}_{\nu }(-(-G)^{\nu }t^{\nu })$

Table 1 sums up the main facts we have discussed on Markov, semi-Markov and para-Markov chains. Note that for $\nu =1$ semi-Markov and para-Markov chains reduce to Markov ones. Once again, we stress the fact that the governing equation of Markov chains is driven by the first derivative, which is a local operator, whereas the governing equations of the semi-Markov and para-Markov chains depend on the Caputo derivative of order $\nu$, which is a non-local operator. It is related to the characteristic of the processes themselves: the probability of a future state depends both on the present value of the process and also on the past.

## Data Availability

Data and programs concerning this paper can be found at https://github.com/Lorenzo-Facciaroni/Exchangeable-fractional-Poisson.

## References

[1] Arendt, W., Batty, C., Hieber, M., Neubrander, F.: Vector Valued Laplace Transform and Cauchy Problems. Birkhäuser, Basel (2010)

[2] Ascione, G., Leonenko, N., Pirozzi, E.: Fractional immigration death processes. J. Math. Anal. Appl. **495**, 1284–1323 (2021)

[3] Baeumer, B., Meerschaert, M.M.: Stochastic solutions for fractional Cauchy problems. Fract. Calc. Appl. Anal. **4**(4), 481–500 (2001)

[4] Barlow, R.E., Mendel, M.B.: de Finetti-type representations for life distributions. J. Am. Stat. Assoc. **87**(420), 1116–1122 (1992)

[5] Beghin, L., Orsingher, E.: Fractional Poisson processes and related planar random motions. Electron. J. Probab. **14**(61), 1790–1826 (2009)

[6] Beghin, L., Ricciuti, C.: Time-inhomogeneous fractional Poisson processes defined by the multistable subordinator. Stoch. Anal. Appl. **37**(2), 171–188 (2019)

[7] Caramellino, L., Spizzichino, F.: Dependence and aging properties of lifetimes with Schur-constant survival functions. Probab. Eng. Inf. Sci. **8**(1), 103–111 (1994)

[8] Caramellino, L., Spizzichino, F.: WBF property and stochastical monotonicity of the Markov process associated to Schur-constant survival functions. J. Multivar. Anal. **56**(1), 153–163 (1996)

[9] Chi, Y., Yang, J., Qi, Y.: Decomposition of a Schur-constant model and its applications. Insur. Math. Econ. **44**(3), 398–408 (2009)

[10] Comtet, L.: Advanced Combinatorics: The Art of Finite and Infinite Expansions. Springer, New York (2012)

[11] De Gregorio, A., Iafrate, F.: Telegraph random evolutions on a circle. Stoch. Process. Appl. **141**, 79–108 (2021)

[12] Di Crescenzo, A., Martinucci, B., Meoli, A.: A fractional counting process and its connection with the Poisson process. ALEA **13**, 291–307 (2016)

[13] Faà di Bruno, F.: Sullo sviluppo delle funzioni. Annali di Scienze Matematiche e Fisiche **6**, 479–868 (1855)

[14] Garra, R., Orsingher, E., Polito, F.: State-dependent fractional point processes. J. Appl. Probab. **52**(1), 18–36 (2015)

[15] Garrappa, R., Popolizio, M.: Computing the matrix Mittag-Leffler function with applications to fractional calculus. J. Sci. Comput. **77**, 129–153 (2017)

[16] Georgiou, N., Kiss, I.Z., Scalas, E.: Solvable non-Markovian dynamic network. Phys. Rev. E **92**(4), 171–188 (2015) 10.1103/PhysRevE.92.042801 26565283

[17] Gershgorin, S.A.: Über die Abgrenzung der Eigenwerte einer Matrix. News Russ. Acad. Sci. Math. Ser. **6**, 749–754 (1931)

[18] Gupta, N., Kumar, A.: Fractional Poisson processes of order and beyond. J. Theor. Probab. **36**(4), 2165–2191 (2023)

[19] Higham, N.J.: Functions of Matrices: Theory and Computation. SIAM, Philadelphia (2008)

[20] Horn, R., Johnson, C.R.: Matrix Analysis. Cambridge University Press, Cambridge (2012)

[21] James, L.F.: Lamperti-type laws. Ann. Appl. Probab. **20**(4), 1303–1340 (2010)

[22] Kataria, K.K., Vellaisamy, P.: On distributions of certain state-dependent fractional point processes. J. Theor. Probab. **32**, 1554–1580 (2019)

[23] Kolokoltsov, V.: Generalized continuous-time random walks, subordination by hitting times, and fractional dynamics. Theory Probab. Appl. **53**(4), 594–609 (2009)

[24] Lamperti, J.: An occupation time theorem for a class of stochastic processes. Trans. Am. Math. Soc. **88**, 380–387 (1958)

[25] Laskin, N.: Fractional Poisson process. Commun. Nonlinear Sci. Numer. Simul. **8**, 201–213 (2003)

[26] Leonenko, N., Scalas, E., Trinh, M.: The fractional non-homogeneous Poisson process. Statist. Probab. Lett. **120**(4), 147–156 (2017)

[27] Maheshwari, A., Vellaisamy, P.: Fractional Poisson process time-changed by Lévy subordinator and its inverse. J. Theor. Probab. **32**(4), 1278–1305 (2019)

[28] Mainardi, F., Gorenflo, R., Scalas, E.: A fractional generalization of the Poisson processes. Vietnam J. Math. **32**(SI), 53–64 (2004)

[29] Mainardi, F., Gorenflo, R., Scalas, E.: Uncoupled continuous-time random walks: solution and limiting behavior of the master equation. Phys. Rev. E **69.1**(1), 153–163 (2004) 10.1103/PhysRevE.69.011107 14995604

[30] Meerschaert, M., Toaldo, B.: Relaxation patterns and semi-Markov dynamics. Stoch. Process. Appl. **129**(9), 2850–2879 (2019)

[31] Meerschaert, M., Nane, E., Vellaisamy, P.: The fractional Poisson process and the inverse stable subordinator. Electron. J. Probab. **129**(9), 2850–2879 (2019)

[32] Metzler, R., Klafter, J.: The random walk’s guide to anomalous diffusion: a fractional dynamics approach. Phys. Rep. **339**(1), 1–77 (2000)

[33] Norris, J.R.: Markov Chains. Cambridge University Press, Cambridge (1998)

[34] Orsingher, E., Polito, F.: Fractional pure birth processes. Bernoulli **16**(3), 858–881 (2010)

[35] Orsingher, E., Polito, F.: Randomly stopped nonlinear fractional birth processes. Stoch. Anal. Appl. **31**(2), 262–292 (2013)

[36] Orsingher, E., Ricciuti, C., Toaldo, B.: On semi-Markov processes and their Kolmogorov’s integro-differential equations. J. Funct. Anal. **275**(4), 830–868 (2018)

[37] Prabhakar, T.R.: A singular integral equation with a generalized Mittag-Leffler function in the kernel. Yokohama Math. J. **19**(1), 7–15 (1971)

[38] Ricciuti, C., Toaldo, B.: Semi-Markov models and motion in heterogeneous media. J. Stat. Phys. **169**(2), 340–361 (2017)

[39] Ricciuti, C., Toaldo, B.: From semi-Markov random evolutions to scattering transport and superdiffusion. Commun. Math. Phys. **401**(3), 2999–3042 (2023)

[40] Scalas, E., Toaldo, B.: Limit theorems for prices of options written on semi-Markov processes. Theory Probab. Math. Stat. **105**(3), 3–33 (2021)

[41] Tuncel, A., Aslan, T.A.: Some bivariate Schur-constant distributions and application to life insurance. J. Comput. Appl. Math. **457**, 116296 (2025)

